# Targeting myomiRs by tocotrienol-rich fraction to promote myoblast differentiation

**DOI:** 10.1186/s12263-018-0618-2

**Published:** 2018-11-29

**Authors:** Azraul Mumtazah Razak, Shy Cian Khor, Faizul Jaafar, Norwahidah Abdul Karim, Suzana Makpol

**Affiliations:** Department of Biochemistry, Faculty of Medicine, Level 17, Preclinical Building, Universiti Kebangsaan Malaysia Medical Centre (UKMMC), Jalan Yaakob Latif, Bandar Tun Razak, Cheras, 56000 Kuala Lumpur, Malaysia

**Keywords:** Muscle, Myoblast, MicroRNA, Gene expression, Tocotrienol, Cell differentiation

## Abstract

**Background:**

Several muscle-specific microRNAs (myomiRs) are differentially expressed during cellular senescence. However, the role of dietary compounds on myomiRs remains elusive. This study aimed to elucidate the modulatory role of tocotrienol-rich fraction (TRF) on myomiRs and myogenic genes during differentiation of human myoblasts. Young and senescent human skeletal muscle myoblasts (HSMM) were treated with 50 μg/mL TRF for 24 h before and after inducing differentiation.

**Results:**

The fusion index and myotube surface area were higher (*p* < 0.05) on days 3 and 5 than that on day 1 of differentiation. Ageing reduced the differentiation rate, as observed by a decrease in both fusion index and myotube surface area in senescent cells (*p* < 0.05). Treatment with TRF significantly increased differentiation at days 1, 3 and 5 of young and senescent myoblasts. In senescent myoblasts, TRF increased the expression of *miR-206* and *miR-486* and decreased *PTEN* and *PAX7* expression*.* However, the expression of *IGF1R* was upregulated during early differentiation and decreased at late differentiation when treated with TRF. In young myoblasts, TRF promoted differentiation by modulating the expression of *miR-206*, which resulted in the reduction of *PAX7* expression and upregulation of *IGF1R*.

**Conclusion:**

TRF can potentially promote myoblast differentiation by modulating the expression of myomiRs, which regulate the expression of myogenic genes.

## Background

Satellite cells, which are located between the basal lamina and sarcolemma, act as vital components of the skeletal muscle tissue as they possess regeneration capacity. These satellite cells are mitotically quiescent and arrested at the G_0_ phase. These cells express a limited number of genes and proteins [1]. In response to stress, such as muscle injury or physiological change, satellite cells are activated and undergo myogenesis, which involves a series of processes [2]. These cells migrate to the damaged site and withdraw from the G_0_ phase to re-enter the cell cycle progression. The cells then undergo proliferation, differentiation and subsequently fuse with the adjacent muscle fibre to form a new muscle fibre [3]. In this proliferating state, the satellite cells are known as myoblast cells. Ageing gradually reduces the regenerative capacity of skeletal muscles resulting a decrease in the muscle mass and strength [4]. This contributes to age- or injury-induced muscle weakness leading to frailty in the elderly, which is one of the major health problems.

The myogenic program is controlled by various transcription factor families such as paired box gene family consisting of PAX3 and PAX7 and myogenic regulator family including MYOD1, MYOG, Myf5 and Myf6 [1, 3]. The PAX7 transcription factor is required for muscle satellite cell biogenesis and specification of the myogenic precursor lineage [5]. Functioning upstream of the MYOD family, PAX7 is expressed in proliferating myoblasts, but is rapidly downregulated during differentiation. In mice, loss of PAX7 expression resulted in the differentiation of satellite cells into fibroblasts instead of myoblasts [6]. Most of the activated satellite cells proliferate, downregulate PAX7 and promote MYOD to progress into differentiation. Various growth factors and hormones such as insulin-like growth factor (IGF) [7], myostatin and follistatin [8], leukaemia inhibitory factors [9], hepatocyte growth factors and neuronal nitric oxide synthase are involved in muscle hypertrophy [10]. All of these modulators activate several pathways that modulate the expression of myogenic transcription factors.

MicroRNAs (miRNAs) have gained tremendous attention and provide a new avenue for understanding the regulatory mechanism of skeletal muscle development. miRNAs are evolutionary conserved small RNAs that have been identified as post-transcriptional regulators to suppress the expression of target genes. The suppression of gene expression is mediated by the binding of miRNA to the 3′ untranslated region (UTR) of the target mRNA [11]. miRNAs have been found to be involved in the regulation of various pathways that contribute to the modulation of several diseases, as a single miRNA can target several mRNAs. miRNAs are expressed in specific tissues, and those miRNAs that are specifically expressed in striated muscles are known as myomiRs [12]. Several myomiRs have been identified, including *miR-1*, *miR-133a*, *miR-133b*, *miR-206*, *miR-486* and *miR-499* [11]. Each myomiR has its own specific or overlapping target mRNA, which functions in promoting myoblast proliferation and differentiation and is differentially expressed during myogenesis. During the differentiation of myoblasts, the expression of *miR-133b*, *miR-206* and *miR-486* is elevated, resulting in the downregulation of *PAX7* mRNA that promotes myogenic differentiation [5]. Thus, miRNAs play an irreplaceable role in the regulation of skeletal muscle differentiation.

Elucidation of the involvement of myomiRs during differentiation of human satellite cells provides current information of possible interactions between transcription factors, myomiRs and their target mRNAs, especially when modulated by dietary compounds. We proposed vitamin E as a promising agent to modulate the expression of myomiRs. Vitamin E consists of α-, β-, γ- and δ-tocopherol and α-, β-, γ- and δ-tocotrienol, all of which are potent lipid-soluble antioxidants. Vitamin E supplements have been found to prevent muscle damage [13]. However, the molecular mechanism of vitamin E in modulating muscle health remains elusive. Besides the tocopherol isomer, a mixture of tocotrienols particularly known as tocotrienol-rich fraction (TRF), which are less studied shows a better effect compared to the tocopherol single isomer [14]. TRF is commonly extracted from palm oil and consists of α-tocopherol and α-, β-, γ- and δ-tocotrienol. It has been reported to protect against oxidative damage and suppress reactive oxygen species (ROS) production [15]. A previous study showed that TRF prevents the replicative senescence of myoblast cells and promotes myogenic differentiation in which its activity is higher than the tocopherol isomer [16]. Interestingly, another study found that TRF prevents replicative senescence of fibroblasts by inhibiting the expression of *miR-34a* and increasing the expression of *CDK4* [17]. As TRF is known to modulate miRNA expression, this study aimed to elucidate its modulatory role on myomiRs and myogenic genes during the differentiation of human myoblasts.

## Materials and methods

### Cell culture and serial passaging

The Clonetics® Skeletal Muscle Myoblast Cell System containing normal human skeletal muscle myoblasts (HSMM; catalogue no. CC-2580, lot 0000257384), sourced from the quadriceps muscle of a 17-year-old female cadaver, was purchased from Lonza, USA. Cells were maintained in the growth medium, Skeletal Muscle Growth Media-2 (SkGM™-2 Medium) that consisted of SkBM™-2 Basal medium. The SkGM™-2 SingleQuots™ Kit [catalogue no. CC-3244 containing human epidermal growth factor (hEGF), dexamethasone, l-glutamine, foetal bovine serum (FBS) and gentamicin/amphotericin B (GA)]. Cell populations were trypsinised when they reached 70 to 80% confluency. For passaging, the culture medium was warmed to 37 °C and the cells were seeded at 5000–7500 cells/cm^2^ and were incubated at 37 °C in a humid atmosphere containing 5% carbon dioxide (CO_2_). At each passage, the number of divisions was calculated as log(N/n)/log 2, where N is the number of cells at the time of passage and n is the number of cells initially plated. The cells were divided into 2 groups, young cells with population doubling 14 (MPD 14) and senescent cells with population doubling 21 (MPD 21) [18].

### Induction of differentiation

Differentiation medium was prepared by adding 2% horse serum to DMEM-F12 medium. To induce differentiation, both the groups of cells were plated at 20,000 cells/cm^2^ in 24-well polystyrene cell culture plates (Thermo Fisher™ Nunc™, Waltham, USA) and incubated overnight in a growth medium in a cell culture incubator (37 °C, 5% CO_2_). The following morning, the growth medium was replaced with differentiation medium and for the TRF-treated groups, both young and senescent cells were treated with 50 μg/mL of TRF groups) [18]. The cultures were then incubated for 5 days.

### Determination of myogenic purity

The myogenic purity of the cultures was monitored by determining the expression of desmin, a cytoskeletal protein that is expressed only in myogenic cells and not in fibroblasts. The number of desmin-positive cells, represented as a percentage of the total number of nuclei, was determined as the myogenic purity of the cell culture, and at least 500 cells were counted. Immunocytochemistry was performed using an antibody specific for desmin, at a dilution of 1:50 (clone D33; DAKO, Denmark). The cells were washed with × 1 phosphate-buffered saline (PBS) and fixed with 100% ethanol for 10 min. The fixation agent was removed by washing three times with × 1 PBS for 5 min. Non-specific binding sites were blocked with 1% FBS diluted in PBS for 30 min. The cells were then incubated with primary antibody against desmin. Specific antibody binding was detected using Alexa Fluor 488 (Invitrogen, USA) directly coupled to the secondary antibody at a dilution of 1:500. The nuclei were fluorescently detected by Hoechst staining (Sigma, USA) at a dilution of 0.0001% *w*/*v*. All images were digitalised using ImageJ software.

### Quantification of the surface area of myotubes and myonuclei

Positive fluorescent areas of five randomly chosen fields from three individual experiments were evaluated. For each treatment, the mean area of the untreated group was used to calculate the percent increase or decrease in the area of myotubes.

### Quantification of fusion index

To calculate the fusion index, the number of nuclei incorporated into the myotubes (> 2 nuclei) were counted and the ratio of this number to the total number of nuclei was determined.

### TRF preparation and treatment

Gold Tri E 70 (Sime Darby Bioganic Sdn. Bhd., Malaysia) was used in this study. This Gold Tri E 70 consists of 25% α-tocopherol and 75% tocotrienol. Further, HPLC analysis of Gold Tri E 70 revealed that it consisted of 173.6 mg/g α-tocopherol, 193.4 mg/g α-tocotrienol, 26.2 mg/g β-tocotrienol, 227.7 mg/g γ-tocotrienol and 98.2 mg/mg δ-tocotrienol. TRF stock solution was freshly prepared in the dark by dissolving 1 g of Gold Tri E 70 (Sime Darby Bioganic Sdn. Bhd., Malaysia) in 1 mL of 100% ethanol (1:1) and stored at − 20 °C for not more than 1 month. TRF was activated by incubating 45 μL of TRF stock solution (1 g/1 mL) with 60 μL of FBS, overnight at 37 °C. To prepare TRF at a concentration of 50 μg/mL, 90 μL of DMEM with 10% FBS and 105 μL of 100% ethanol were added to the activated TRF, after which 600 μL of the mixture containing FBS and 100% ethanol (1:1) was added. The TRF solution (50 μg/mL) was prepared using the culture medium. Myoblasts were treated with 50 μg/mL TRF for 24 h, and untreated myoblasts were incubated with SKGM-2 medium (Lonza, USA) for proliferation analysis and with DMEM-F12 medium (Lonza, USA) for differentiation analysis. A series of dosage titrations performed in a previous study showed that 50 μg/mL of TRF treatment for 24 h produced the highest percentage of viable young and senescent myoblasts [16]. Furthermore, the myoblast cells used in the present study are similar to the ones in our previous study [16]. The media for both untreated and TRF-cells were changed simultaneously, and both groups of cells were harvested on the same day.

### Primer design

Forward primers for microRNA were designed according to the miRNA sequences listed in the miRBase database (http://www.mirbase.org). For *miR-486*, *miR-486-5p* form was selected for forward primer synthesis. Table 1 shows the forward primer sequences for validated miRNAs. Primers for human *GAPDH*, *PAX7*, *IGF1R* and *PTEN* were designed from listed NIH GenBank database using Primer 3 software and blasted with sequences in the GenBank database for specificity confirmation. The efficiency and specificity of each primer set was confirmed by melting profile evaluation. The primer sequences for quantitative gene expression analysis are shown in Table 2.

**Table 1:** Primer sequences of validated miRNAs

Access no.	miRBase ID	Mature miRNA sequence (5′➔3′)	Size (bp)
miRBase (MIMAT)			
MIMAT0002177	hsa-miR-486-5p	UCCUGUACUGAGCUGCCCCGAG	22
MIMAT0000462	hsa-miR-206	UGGAAUGUAAGGAAGUGUGUGG	22
MIMAT0000770	hsa-miR-133b	UUUGGUCCCCUUCAACCAGCUA	22
NCBI			
NR_002752	*RNU6B*	CGCAAGGAUGACACGCAAAUUCGUGAAGCGUUCCAUAUUUUU	42

**Table 2:** Primer sequences for quantitative gene expression analysis

Gene	Primer	Primer sequence (5′➔3′)	PCR product size (bp)
*GAPDH*	Forward	TCCCTGAGCTGAACGGGAAG	217
	Reverse	GGAGGAGTGGGTGTCGCTGT	
*PTEN*	Forward	ACTTGAAGGCGTATACAGGACCA	199
	Reverse	AATGTCTTTCAGCACAAAGATTGTA	
*PAX7*	Forward	GTGCCCTCAGTGAGTTCGAT	118
	Reverse	GTTCCG ACI CCACATCCGAG	
*IGF1R*	Forward	TGGAGTGCTGTATGCCTCTG	153
	Reverse	CCCTTGGCAACTCCTTCATA	

### RNA extraction

Total RNA was extracted from different treatment groups of myoblasts using TRI Reagent (Molecular Research Center, Cincinnati, USA) according to the manufacturer’s instructions. Polyacryl carrier (Molecular Research Center, Cincinnati, USA) was added to each extracted sample to precipitate the total RNA. Extracted RNA pellet was washed with 75% ethanol and dried prior to dissolving it in RNase-free distilled water. Aliquots of total RNA were stored at − 80 °C immediately after extraction. The yield and purity of the extracted total RNA was determined by nanodrop spectrophotometer (Thermo Scientific, USA).

### Real-time qRT-PCR

For quantitative analysis of miRNAs, reverse transcription (RT) was first performed with 10 ng of total RNA using Taqman microRNA Reverse Transcription kit (Applied Biosystems, USA) according to the manufacturer’s instructions. PCR reactions were then performed to quantitate the expression levels of myomiRs (miR-206, miR-133b and miR-486) using Taqman Universal PCR Master Mix No AmpErase UNG (Applied Biosystems, USA), according to manufacturer’s instructions, and Taqman microRNA assay kit (Applied Biosystems, USA) was used for the detection of myomiRs of interest. PCR amplification was performed in iQ5 Multicolor Real-Time PCR iCycler (Bio-Rad, USA) at 95 °C for 10 min, followed by 40 cycles of 95 °C for 15 s and 60 °C for 60 s. PCR reactions were performed in triplicates. The expression level of all myomiRs was normalised to the expression of RNU6B. The relative expression value (REV) of miRNAs was calculated using the equation for 2^−ΔCt^ method of relative quantification [16, 19]:

$$ \mathrm{REV}={2}^{\mathrm{Ct}\ \mathrm{value}\ \mathrm{of}\ \mathrm{RNU}6\mathrm{B}-\mathrm{Ct}\ \mathrm{value}\ \mathrm{of}\ \mathrm{miRNA}} $$

Expression of *PAX7*, *IGF1R* and *PTEN* genes was analysed using KAPA SYBR Fast One Step qRT-PCR kit (KAPA Biosystems, USA) and iQ5 Multicolor Real-Time PCR iCycler (Bio-Rad, USA). Each qRT-PCR mixture contained 11.7 μL nuclease-free water, 10 μL KAPA SYBR Fast master mix, 0.3 μL RT enzyme, 1 μL of 100 μM forward primer, 1 μL of 100 μM reverse primer and 1 μL total RNA (50–100 ng). Reactions were performed in iQ5 Multicolor Real-Time PCR iCycler (Bio-Rad, USA) at 42 °C for 5 min and 95 °C for 4 min, followed by 40 cycles of 95 °C for 3 s and 60 °C for 20 s. qRT-PCR reactions were performed in triplicates. *GAPDH* was used as a normalisation reference gene [19, 20]. The relative expression value (REV) of the genes of interest was calculated using equation for the 2^−ΔCt^ method of relative quantification [16, 19]:

$$ \mathrm{REV}={2}^{\mathrm{Ct}\ \mathrm{value}\ \mathrm{of}\ \mathrm{GAPDH}-\mathrm{Ct}\ \mathrm{value}\ \mathrm{of}\ \mathrm{the}\ \mathrm{gene}\ \mathrm{of}\ \mathrm{interest}} $$

### Determination of cell cycle profile

Untreated control and TRF-treated myoblasts were sub-cultured in 10 cm^2^ tissue culture dish. After 24 h of incubation, cells were harvested and prepared for cell cycle analysis using CycleTEST PLUS DNA Reagent Kit (Becton Dickinson, USA) according to the manufacturer’s instructions. The cell cycle status was analysed by FACS Calibur flow cytometer (Becton Dickinson, USA) using propidium iodide (PI) as a specific fluorescent dye probe. The PI fluorescence intensity of 15,000 cells was measured for each sample.

### Statistical analysis

Data are presented as the mean ± SD. Experiments were performed at least three times, and data were analysed by Student’s *t* test and one-way analysis of variance (ANOVA). Significance was accepted at *p* < 0.05.

## Results

### Effects of TRF on the morphology and myogenic purity of skeletal muscle myoblasts

**Fig. 1:**
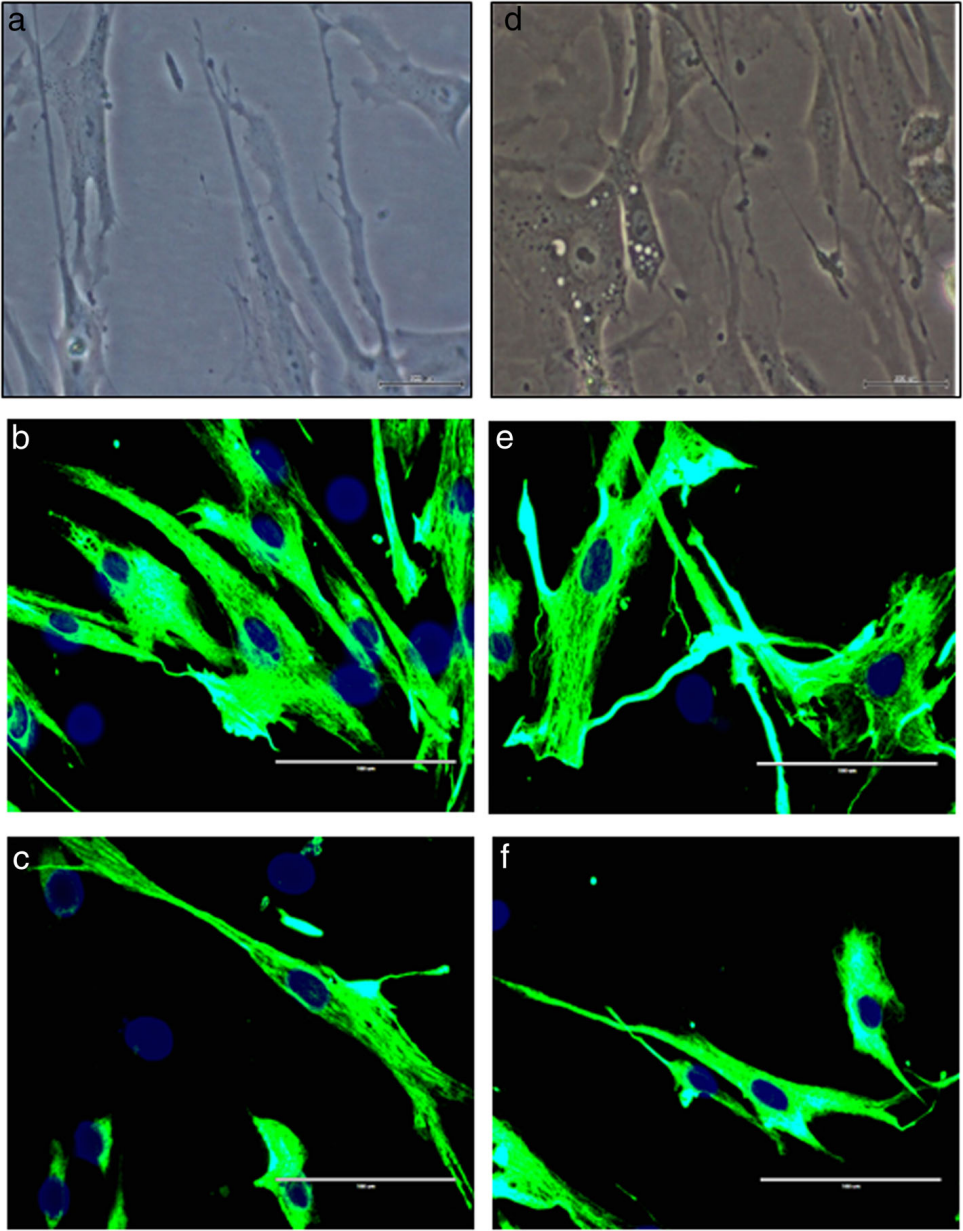
Morphology of young and senescent myoblasts for control and TRF-treated cells. Observation was carried out under phase contrast (**a**, **d**) and fluorescence microscopy (**b**, **c**, **e**, **f**) (× 40 magnification). The myoblast cells were stained with an antibody against desmin (green), and the nuclei were stained with Hoechst (blue). Control senescent myoblasts appeared larger and flatter with the presence of more prominent intermediate filaments (**d**, **e**) compared to control young myoblasts (**a**, **b**). Some of the TRF-treated senescent myoblasts (**f**) remained spindle-shaped which resembled young control while some exhibited flatter and larger morphology. No morphological changes was observed for TRF-treated young myoblasts (**c**)

Young myoblasts (PD 14) exhibited normal spindle shape with round nuclei (Fig. 1a, b, c), while senescent myoblasts were larger and flatter and consisted of prominent intermediate filaments (Fig. 1d, e). Senescent myoblasts exhibited different morphological features when treated with TRF. Most of the cells were spindle-shaped (Fig. 1f), which resembled the TRF-treated young myoblasts (Fig. 1c). The myogenicity of the myoblasts was more than 90% in both treatment groups (Table 3). A comparison between different treatment groups showed that the myogenicity was similar in all treatment groups.

**Table 3:** Myogenic purity of myoblasts in culture

Groups	Myogenicity (%)
Young control (PD14)	95.23 ± 2.14
Senescent control (PD21)	92.84 ± 0.80
Young TRF-treated	96.35 ± 1.87
Senescent TRF-treated	94.22 ± 0.42

### Effects of TRF on differentiation analysis of skeletal muscle myoblasts

**Fig. 2:**
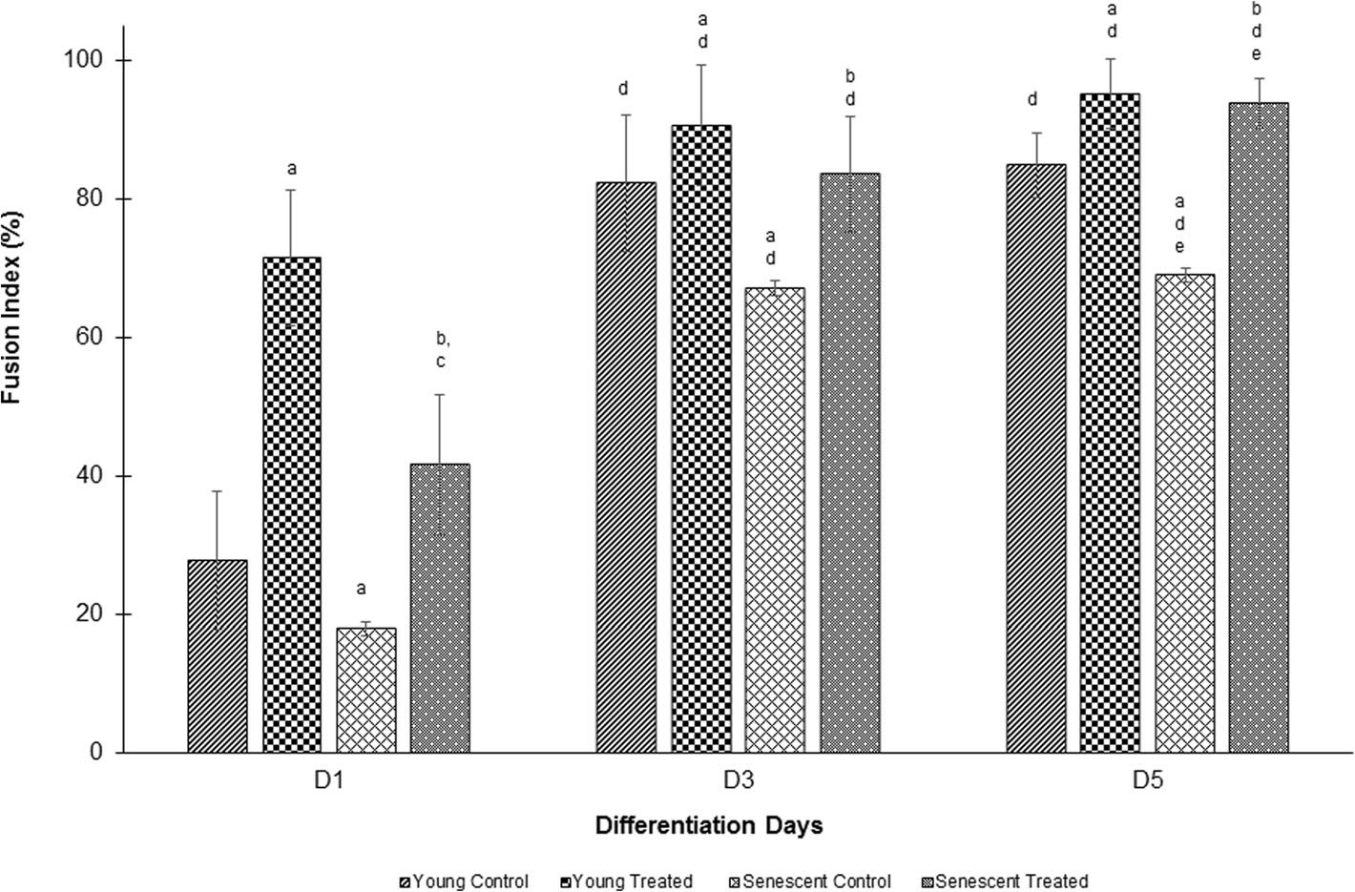
Effect of TRF on myoblasts differentiation. Fusion index was measured as an index of differentiation. ^a^Denotes *p* < 0.05 compared to young control, ^b^*p* < 0.05 compared to senescent control, ^c^*p* < 0.05 compared to young treated, ^d^*p* < 0.05 compared to day 1 of the same treatment and ^e^*p* < 0.05 compared to day 3 of the same treatment. Data are presented as mean ± SD, n = 3

**Fig. 3:**
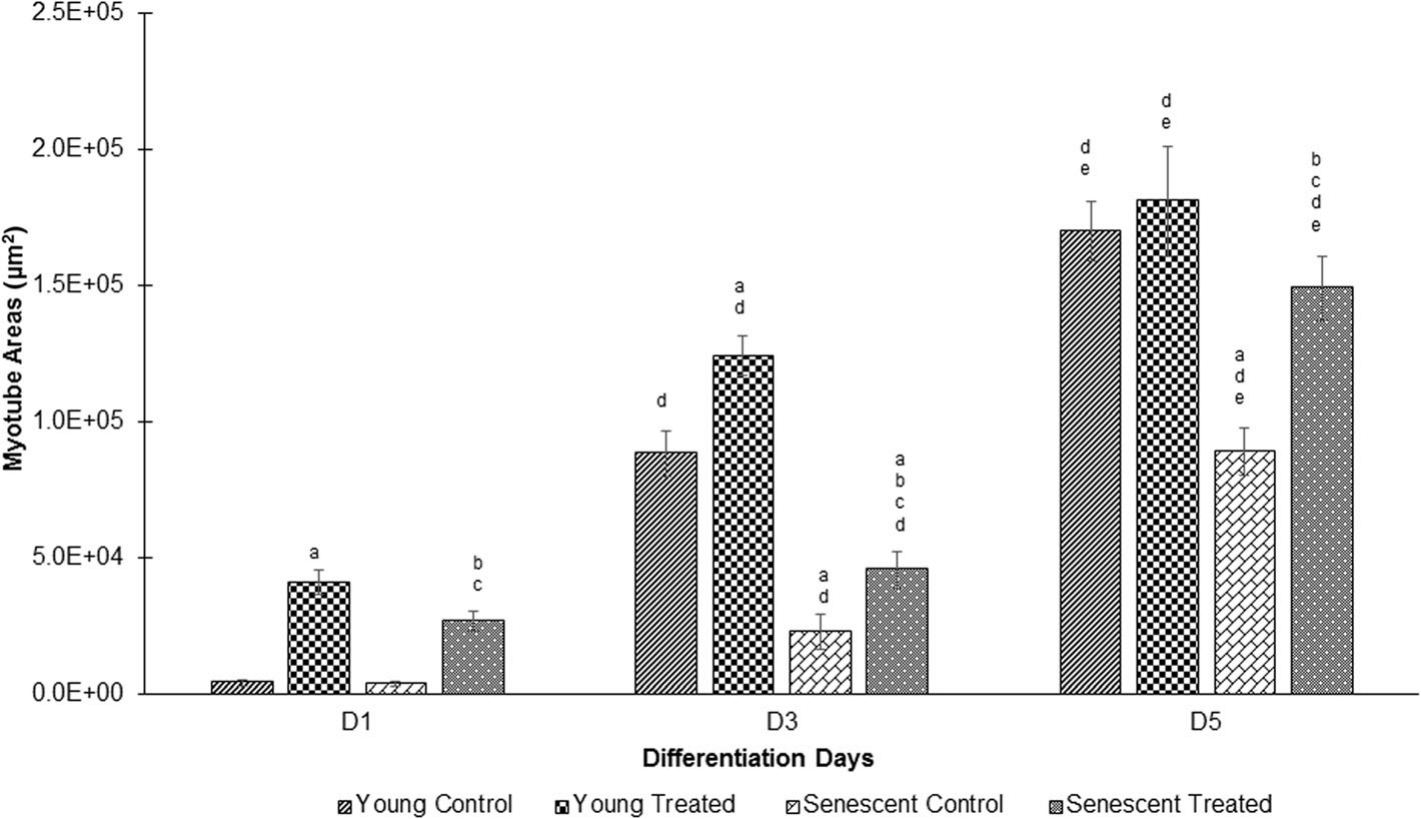
Effect of TRF on myoblasts differentiation as measured by myotube surface area. ^a^Denotes *p* < 0.05 compared to young control, ^b^*p* < 0.05 compared to senescent control, ^c^*p* < 0.05 compared to young treated, ^d^*p* < 0.05 compared to day 1 of the same treatment and ^e^*p* < 0.05 compared to day 3 of the same treatment. Data are presented as mean ± SD, n = 3

The fusion index (Fig. 2) and myotube surface area (Fig. 3) were greater on days 3 and 5 than that on day 1 of differentiation. Ageing causes a significant reduction in the differentiation rate of senescent myoblasts on days 3 and 5 as compared to that of young myoblasts (control) (*p* < 0.05), as observed by decreased fusion index and surface area of myotubes. Treatment with TRF significantly increased the differentiation rate with an increase in the fusion index and myotube surface area on days 1, 3 and 5 in both young and senescent myoblasts (*p* < 0.05).

### TRF treatment modulates myomiR expression

**Fig. 4:**
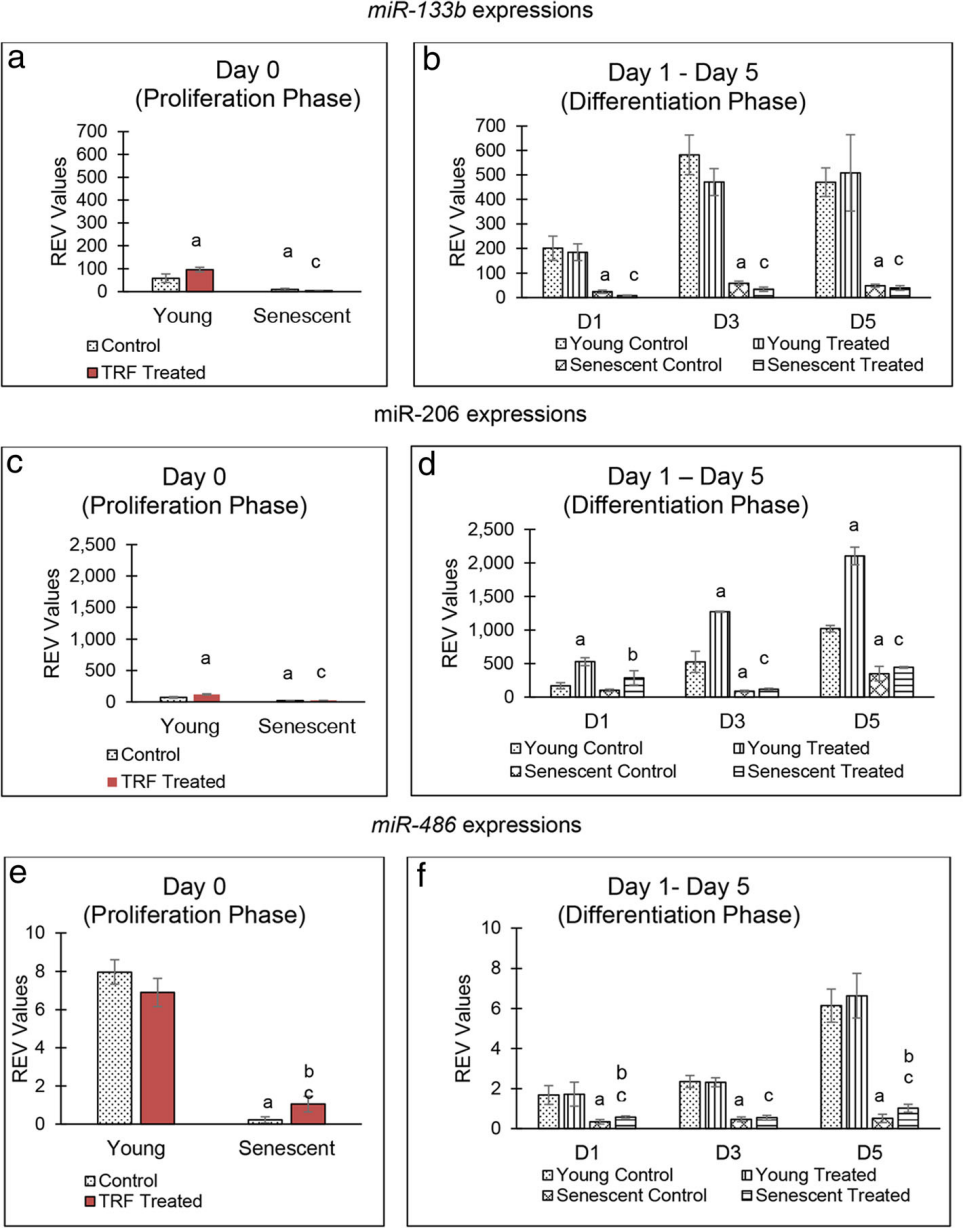
Effect of TRF on the expression of micro RNAs during proliferation and differentiation of young and senescent myoblasts. Expression of *miR-133b* (**a**, **b**), *miR-206* (**c**, **d**) and *miR-486* (**e**, **f**) in young control myoblasts, TRF-treated young myoblasts, senescent control myoblasts and TRF-treated senescent myoblasts. ^a^Denotes *p* < 0.05 compared to young control, ^b^*p* < 0.05 compared to senescent control and ^c^*p* < 0.05 compared to young TRF-treated myoblasts. Data are presented as relative expression value (REV) normalised to *RNU6B* expression (mean ± SD, n = 3)

Changes in the expression of miRNAs were observed in all groups of myoblasts. *miR-133b* expression was reduced significantly in senescent myoblasts during proliferation phase (Fig. 4a). However, there was a significant increase in the expression of *miR-133b* in young myoblasts when treated with TRF (*p* < 0.05). During the differentiation phase, *miR-133b* expression was decreased in senescent myoblasts (*p* < 0.05). No significant change was observed in *miR-133b* expression when young and senescent myoblasts were treated with TRF during differentiation as compared to control group (Fig. 4b).

*miR-206* expression was reduced significantly in senescent myoblasts during the proliferation phase (Fig. 4c). Upon TRF treatment, the expression of *miR-20*6 increased significantly in young myoblasts (*p* < 0.05). During the differentiation phase, *miR-206* expression in senescent myoblasts decreased significantly (*p* < 0.05). However, when treated with TRF, the expression of *miR-206* increased significantly in young myoblasts from day 1 until day 3 of the differentiation phase and increased only on day 1 of differentiation phase in senescent myoblasts (Fig. 4d).

*miR-486* expression was reduced significantly in senescent myoblasts during proliferation and differentiation phases (Fig. 4e, f). However, upon TRF treatment, there was a significant increase in *miR-486* expression in senescent myoblasts during the proliferation phase and on days 1 and 5 during the differentiation phase (*p* < 0.05).

### TRF treatment modulates the expression of target genes and upstream regulators of myomiRs

**Fig. 5:**
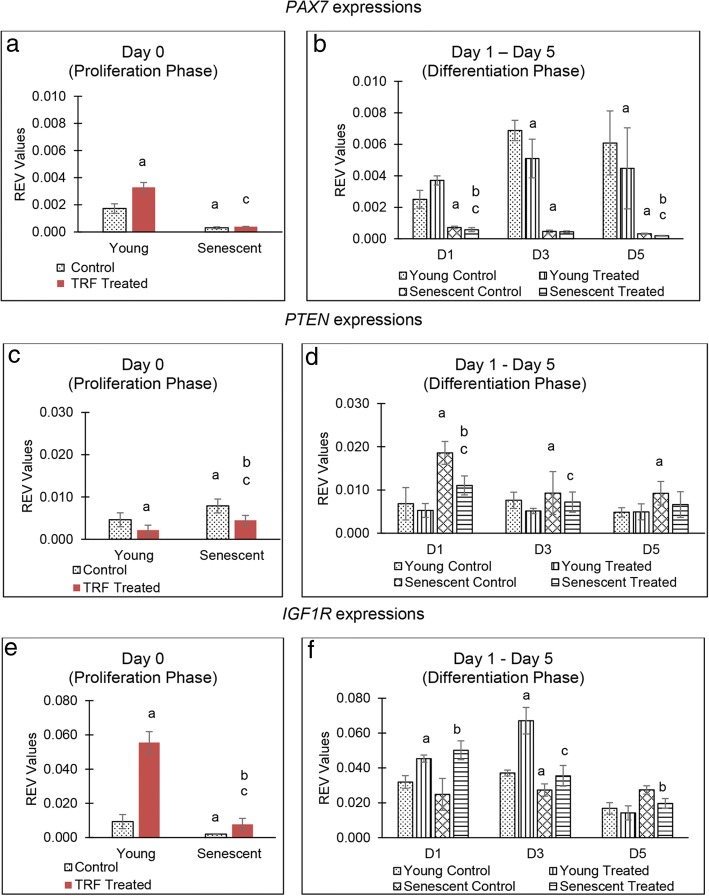
Effect of TRF on the downstream genes expression. Expression of *PAX7* (**a**, **b**), *PTEN* (**c**, **d**) and *IGF1*R (**e**, **f**) in young control myoblasts, TRF-treated young myoblasts, senescent control myoblasts and TRF-treated senescent myoblasts. ^a^Denotes *p* < 0.05 compared to young control, ^b^*p* < 0.05 compared to senescent control and ^c^*p* < 0.05 compared to young treated myoblasts. Data are presented as relative expression value (REV) normalised to *GAPDH* expression (mean ± SD, n = 3)

Senescent myoblasts showed significantly decreased *PAX7* expression during the proliferation phase (*p* < 0.05) (Fig. 5a). Upon TRF treatment, *PAX7* expression was significantly increased in young myoblasts during proliferation. During the differentiation phase, there was a significant reduction in *PAX7* expression in senescent myoblasts (*p* < 0.05). Treatment with TRF during the differentiation phase caused a significant decrease in *PAX7* expression in young myoblasts on days 3 and 5 and on days 1 and 5 in senescent myoblasts (Fig. 5b).

*PTEN* expression increased significantly in senescent myoblasts during proliferation and differentiation phases (*p* < 0.05) (Fig. 5c, d). TRF treatment caused a significant reduction in *PTEN* expression in young and senescent myoblasts during the proliferation phase. During the differentiation phase, treatment with TRF decreased *PTEN* expression in senescent myoblasts on day 1 of differentiation (Fig. 5d).

Senescent myoblasts exhibited significantly decreased *IGF1R* expression during the proliferation phase and on day 3 of the differentiation phase (*p* < 0.05) (Fig. 5e, f). TRF treatment caused a significant increase in *IGF1R* expression in young and senescent myoblasts during the proliferation phase. During the differentiation phase, treatment with TRF caused a significant increase in *IGF1R* expression in senescent myoblasts on day 1, which decreased on day 5 (Fig. 5f).

### Effects of TRF on cell cycle profile

**Fig. 6:**
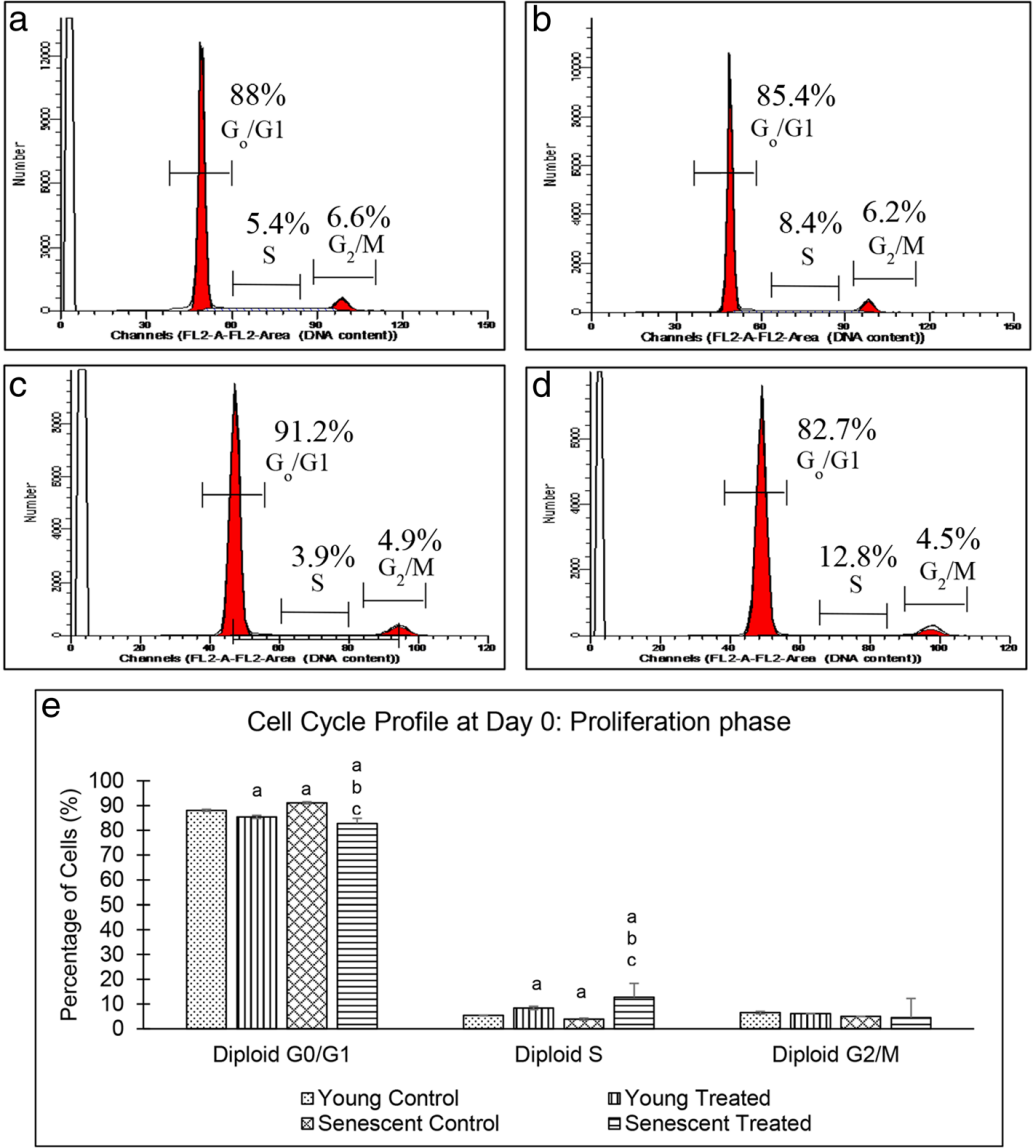
Cell cycle profile of young and senescent myoblasts at day 0 of differentiation. Flow cytometry analysis of cell cycle progression of young control myoblasts (**a**), TRF-treated young myoblasts (**b**), senescent control myoblasts (**c**) and TRF-treated senescent myoblasts (**d**). Quantitative analysis of cell cycle progression of young and senescent myoblasts (**e**). ^a^Denotes *p* < 0.05 compared to young control. ^b^*p* < 0.05 compared to senescent control and ^c^*p* < 0.05 compared to young treated group. Data are expressed as mean ± SD (*n* = 6)

Analysis of cell cycle profile at day 0 showed that myoblast population in the G_0_/G_1_ phase was significantly higher and in the S phase, the population of senescent cells was significantly lower than those in the young cells (*p* < 0.05) (Fig. 6). Treatment with TRF caused a significant reduction in the percentage of senescent myoblasts in the G_0_/G_1_ phase and a significant increase in the percentage of young and senescent myoblasts in the S phase (*p* < 0.05) (Fig. 6e).

**Fig. 7:**
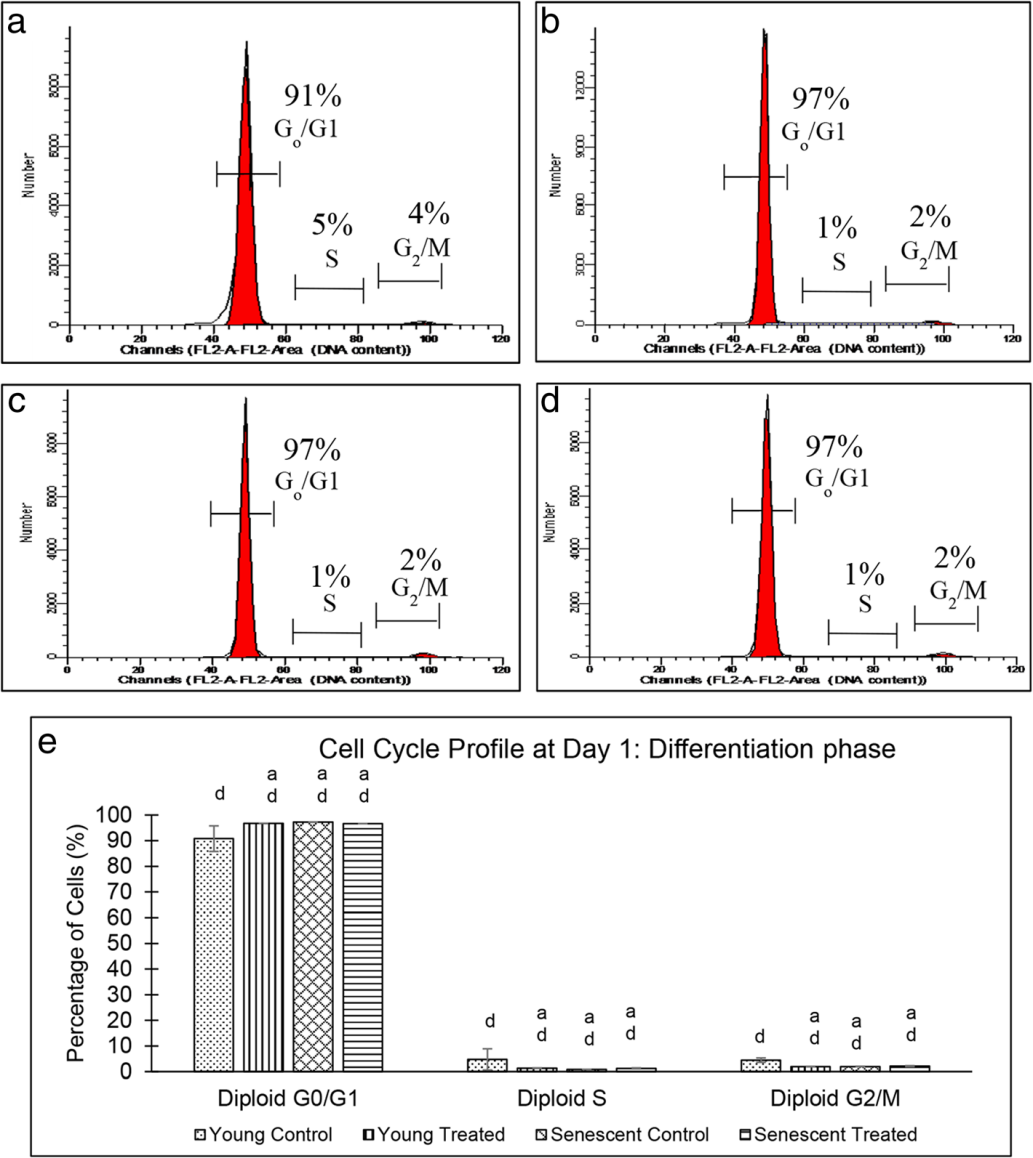
Cell cycle profile of young and senescent myoblasts at day 1 of differentiation. Flow cytometry analysis of cell cycle progression of young control myoblasts (**a**), TRF-treated young myoblasts (**b**), senescent control myoblasts (**c**) and TRF-treated senescent myoblasts (**d**). Quantitative analysis of cell cycle progression of young and senescent myoblasts (**e**). ^a^Denotes *p* < 0.05 compared to young control, and ^d^*p* < 0.05 compared to same treatment at day 0 of differentiation. Data are expressed as mean ± SD (*n* = 6)

On day 1 of differentiation, the percentage of myoblasts in the G_0_/G_1_ phase was significantly higher in senescent cells than that in the young cells (*p* < 0.05), while the percentage of cells in the S and G_2_/M phases was significantly reduced in both groups of myoblasts (Fig. 7). A comparison of the cell cycle profile between days 0 and 1 of differentiation showed a significant difference in the percentage of cells in the G_0_/G_1_, S and G_2_/M phases in both groups (*p* < 0.05).

## Discussion

The integrity of myoblast cell structure as well as the formation of myotubes is maintained by the cytoskeleton, cell membrane and the extracellular matrix (ECM) [21]. Young myoblasts are morphologically spindle-shaped and elongated structures. In contrast, senescent myoblasts manifest morphological changes with a flattened structure and larger cytoplasm. Upon induction of differentiation, myoblasts fuse together to form a multinuclear myotube. Young myoblasts form large-branched myotubes, whereas senescent myoblasts form smaller myotubes [16]. As cells senesce, the level of reactive oxygen species (ROS) increases proportionately. In addition, the level of antioxidants is inversely proportional to the level of ROS throughout the senescence process. Accumulation of ROS in the cells induces oxidative stress, which causes oxidative damage to macromolecules such as DNA, RNA, protein and lipid [22]. Consequently, several pathways and cellular metabolism are altered, which leads to changes in the cytoskeleton, cell membrane and the ECM [23], resulting in phenotypic changes in senescent myoblasts, as observed in the present study. Therefore, introducing antioxidants to the altered system is predicted to reduce the oxidative stress, thus delaying the senescence of myoblasts.

Vitamin E, particularly TRF, plays a pivotal role in scavenging peroxyl radicals and prevents the peroxidation of macromolecules, thus improving the oxidative status of cells [24]. Vitamin E consists of two isomers, tocopherol and tocotrienol. Tocotrienol has been reported to possess better antioxidant activity and effectively reduces the oxidative stress in lipophilic environment [25]. In this study, TRF treatment ameliorated the morphological structure of senescent myoblasts and showed similar features to young myoblasts. A previous study showed that TRF treatment of senescent fibroblast cells reversed the morphological structure to form young fibroblasts [26]. Similarly, in a previous study by Khor et al., it was reported that senescent myoblasts treated with TRF appeared to have similar morphological features as young myoblast cells [16]. This observation could be due to the modulation of protein expression, which is involved in maintaining cell structure. Matrix metalloproteinase (MMP), responsible for the degradation of procollagen, is highly expressed in senescent cells. This protein alters cell structure maintenance in senescent cells [27]. However, TRF increases the expression of procollagen in senescent fibroblast cells [28], hence improving the morphological structure of senescent cells as observed in this study.

Homeostasis between proliferation and differentiation of myoblast cells during myogenesis is tightly regulated to prevent uncontrolled proliferation [29]. In the present study, the percentage of senescent myoblast cell population was higher in the G_0_/G_1_ phase and lower in the S phase than young cells, during proliferation. A similar result was also observed in differentiated senescent myoblasts. Interestingly, TRF treatment of both proliferating young and senescent myoblast cells enhanced the cell cycle progression as the cell population in the G_0_/G_1_ and S phases reduced and increased, respectively. However, during the induction of differentiation, TRF promotes cell cycle withdrawal in young myoblast cells. Like other somatic cells, the proliferation capacity of myoblast cells is limited by replicative senescence due to progressive loss of telomere length [30]. In order to prevent tumour progression, cell cycle checkpoints act as barriers to prevent the replication of damaged DNA whereby cells are arrested at the G_0_/G_1_ phase [29].

All cell cycle checkpoints are regulated by several cyclin-dependent kinases (CDKs) and cyclin proteins. Depending on the stimuli or cell environment such as DNA damage response (telomere shortening), CDK inhibitors such as p16 or p21 are expressed to inhibit the formation of CDK/cyclin complex, thus arresting cell cycle progression at the G_0_/G_1_ phase [31]. Previous studies have shown that TRF treatment of senescent fibroblast cells increased the expression of telomerase and enhanced the elongation of telomere [26]. Furthermore, γ-tocotrienol treatment of senescent cells downregulates p16, cyclin D1 and hypophosphorylated-Rb, all of which are involved in cell cycle arrest [32]. Thus, TRF is postulated to modulate telomerase expression, increase the expression of proteins involved in cell cycle to prevent cell cycle arrest and promote proliferation of myoblasts. Treatment with TRF increased the percentage of myoblast cells in the G_0_/G_1_ phase on day 1 of differentiation induction indicating the promotion of cell cycle arrest and inhibition of cell proliferation for differentiation to occur. This could be due to TRF, which is dependent on cell environment or stimuli. Previous studies have shown that γ- and δ-tocotrienol stimulates the differentiation of osteoblasts, which in turn enhance bone formation [33]. Furthermore, a previous study has shown that combined activity of TRF is much better in promoting myoblast differentiation than single α-tocotrienol treatment [16]. However, complete understanding of the mechanism of TRF in promoting the proliferation and differentiation of myoblasts remains elusive.

At the molecular level, regulation of proliferation and differentiation of myoblast cells during myogenesis is associated with several genes and myomiRs (miR) [11]. During cell renewal, quiescent satellite cells upregulate the expression of PAX7 and downregulate the expression of its myogenic regulatory factor (MRF) gene targets, *MYOD1* and *MYOG.* Expression of PAX7 promotes re-entry of quiescent satellite cells into cell cycle progression and enhances the proliferation of myoblasts [6]. In the present study, the expression of *PAX7* gene was increased in differentiated myoblasts, and this increased expression remained constant after several days of differentiation induction. However, TRF treatment increased the expression of *PAX7* gene during proliferation and downregulated its expression during differentiation. Increased expression of PAX7 followed by suppression of myogenesis inhibitors Id2 and Id3 has been reported to upregulate the expression of MYOD1 and MYOG [34]. MYOD1 is directly involved with the activation of p21, cyclin D3 and Rb expression, which are critical for irreversible cell cycle withdrawal of myoblast cells from the G_0_/G_1_ phase during differentiation and terminal differentiation phases [35].

PAX7 expression is regulated by *miR-206* and *miR-486.* As MYOD1 expression is increased, this transcription factor that has its binding site in the promoter regions of *miR-206* and *miR-486*, facilitates the expression of these two myomiRs [5]. Interestingly, TRF treatment upregulated the expression of *miR-206* during proliferation, and this expression was further upregulated during differentiation. Another myomiR, *miR-486*, was upregulated when treated with TRF in proliferated and differentiated myoblasts. In contrast, TRF treatment did not upregulate the expression of *miR-486* during differentiation. Previous studies have shown that the suppression of the *PAX7* gene by *miR-206* and *miR-486* enhanced the commitment of myoblast cells to differentiate [5]. However, overexpression of PAX7 promotes uncontrolled proliferation [36]. Therefore, in the present study, TRF might play a role in maintaining the proliferation and differentiation of myoblasts by modulating the expression of *PAX7* gene, *miR-206* and *miR-486*, without disturbing the homeostasis of myogenesis.

Various modulators that regulate the activity of satellite cells and utilise various signalling pathways, including the IGF1R/P13K pathway, control myogenesis. This pathway mediates the functions of IGF as both IGF-1 and IGF-2 bind to IGF1R. IGF1R was downregulated during the late differentiation stage due to the presence of *miR-133* response element (MRE) located in the 3′UTR [9, 37]. This would explain the direct effects of *miR-133* towards IGF1R as a negative regulator of PI3K/Akt. *miR-133* downregulates Akt phosphorylation via inhibition of IGF1R protein, which is responsible for glucose metabolism, cell proliferation and apoptosis [37, 38]. A reduction in Akt phosphorylation was observed during the differentiation of C2C12 myoblasts. *miR-133* is important to regulate and balance the activity of IGF in muscle cells. IGF1R was found to be deregulated in rhabdomyosarcoma (RMS) where its expression increased consistently and, hence, is suggested as an initial factor responsible for oncogenic transformation of muscle cells [39]. Prolonged and consistent IGF1R expression resulted in increased proliferation and prevention of the differentiation phase [37].

Akt activation also activates mTOR and inhibits GSK3B, a negative regulator of protein synthesis and muscle growth. PTEN is a PI3K phosphatase that deactivates Akt, inhibiting muscle cell growth and muscle cell survival [40]. Decreased PTEN expression stimulates the PI3K/Akt pathway for the promotion and expression of myogenic transcription factors such as MYOD1, MYOG and Myf5 during myoblast proliferation and differentiation. A previous study reported reduced expression of *miR-486* in Duchenne muscular dystrophy [41] and ageing [42]. *miR-486* acts as a mediator for MYOD1 and regulates the PI3K/Akt pathway. *miR-486* is transcribed from an intron of the *Ank1* gene consisting of 39a exon code for muscle-specific Ank1 protein, which connects the sarcomere to the sarcoplasmic reticulum [43]. The expression of *Ank1* gene transcript is controlled by a promoter site that contains two conserved E-boxes, which interact with MYOD [43]. An increase in the expression of *miR-486* by TRF shows that TRF possesses the ability to delay the ageing phenotype and sarcopenia during ageing.

In the present study, TRF treatment has also been shown to increase the expression of myomiRs in young and senescent myoblasts in both proliferation and differentiation phases. Thus, TRF might play a role in the biogenesis of myomiRs directly or indirectly, which involves several processes [11]. Initially, the myomiR is transcribed in the nucleus as a primary transcript or pri-myomiR with a stem-loop structure. Here, the transcription process is modulated by various transcription factors. The pri-myomiR is subsequently processed to form pre-myomiR by the removal of both the end strands. Later, in the cytoplasm, the pre-myomiR loop is cleaved out and unwound by a helicase to form a single-stranded mature myomiR. These modification processes involve various proteins and may be one of the direct or indirect targets of TRF. At the transcriptional level, *miR-206* is regulated by transcription factor FOXO3a [11]. A previous study showed that TRF treatment increases the expression of *FOXO3a* gene [44]. Another finding also reported that γ- and δ-tocotrienol increased the expression of *FOXO3a* gene [45]. Therefore, TRF is predicted to modulate the biogenesis of myomiR via regulation of its transcription factor.

As there was a decrease in the expression of myomiRs in senescent myoblasts, we proposed another mechanism for TRF-mediated regulation of myomiRs, which may be attributed to its radical-scavenging effect. The RNase III enzyme, Dicer, is responsible for cleaving the loop out of pre-miRNA (a major steps in the biogenesis process) to produce double-stranded mature miRNAs [11]. This enzyme is inhibited by various stress factors including ROS, which is accumulated during ageing [46]. Another finding showed that Dicer expression decreased with increased level of oxidative stress and DNA damage. As TRF effectively reduced the levels of ROS, especially in senescent cells, TRF is suggested to modulate myomiRs by reducing the oxidative stress, which in turn enhances the activity and expression of Dicer. Therefore, TRF may be involved in the biogenesis of myomiRs via modulation of Dicer expression. Hence, to verify the specificity of TRF response on Dicer expression, a further study is required by using other antioxidants or by inhibiting the function of miRNA by anti-miR oligonucleotides.

**Fig. 8:**
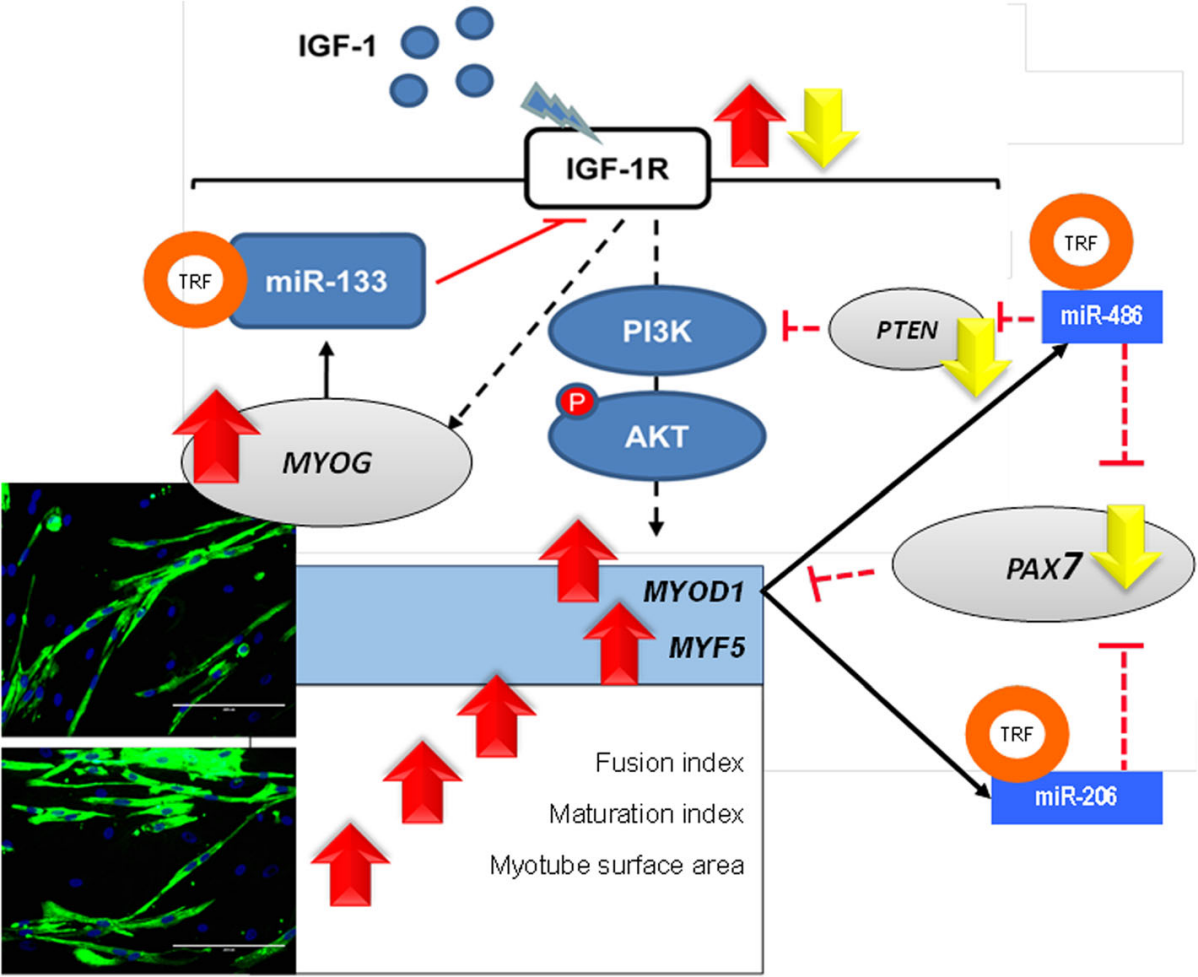
Modulatory effects of TRF on the expression of myomiRs and myogenic regulatory factors

TRF naturally exists as a mixture of various forms of vitamin E; all tocotrienol forms are present and highly concentrated in TRF. However, each cell has its own preference for various forms of vitamin E. A previous study showed that the concentration of α- and δ-tocotrienol was the highest in myoblasts, [18] while γ- and δ-tocotrienol were the most abundant forms in fibroblasts [47]. As previously described, TRF treatment showed a better effect than single isomer treatment. Hence, preferential and selective uptake of vitamin E form by the cell represents the best synergistic effect between vitamin E forms and suitability, which depends on cell environment. Figure 8 summarises the modulatory effect of TRF on the expression of myomiRs and the myogenic regulatory factors. Our results revealed that TRF is a potential muscle differentiation agent that modulates the expression of myomiRs and its target genes involved in myoblast differentiation during myogenesis.

## Conclusion

The findings of the present study demonstrated that tocotrienol-rich fraction with antioxidant and non-antioxidant properties altered the expression of myomiRs, specifically *miR-133b*, *miR-206* and *miR-486*, thereby modifying the expression of their target genes that are involved in myogenesis to promote muscle differentiation in young and senescent myoblasts.
